# Long-term (1990–2019) monitoring of forest cover changes in the humid tropics

**DOI:** 10.1126/sciadv.abe1603

**Published:** 2021-03-05

**Authors:** C. Vancutsem, F. Achard, J.-F. Pekel, G. Vieilledent, S. Carboni, D. Simonetti, J. Gallego, L. E. O. C. Aragão, R. Nasi

**Affiliations:** 1 European Commission, Joint Research Centre, Via E. Fermi 2749–TP 261, I-21027 Ispra (VA), Italy.; 2 CIRAD, UMR AMAP, F-34398 Montpellier, France.; 3 CIRAD, Forêts et Sociétés, F-34398 Montpellier, France.; 4 AMAP, Univ Montpellier, CIRAD, CNRS, INRAE, IRD, Montpellier, France.; 5 GFT Italia Srl, Via Sile 18, Milan, Italy.; 6 National Institute for Space Research (INPE), São José dos Campos, Brazil.; 7 Center for International Forestry Research (CIFOR), Bogor, Indonesia.

## Abstract

Accurate characterization of tropical moist forest changes is needed to support conservation policies and to quantify their contribution to global carbon fluxes more effectively. We document, at pantropical scale, the extent and changes (degradation, deforestation, and recovery) of these forests over the past three decades. We estimate that 17% of tropical moist forests have disappeared since 1990 with a remaining area of 1071 million hectares in 2019, from which 10% are degraded. Our study underlines the importance of the degradation process in these ecosystems, in particular, as a precursor of deforestation, and in the recent increase in tropical moist forest disturbances (natural and anthropogenic degradation or deforestation). Without a reduction of the present disturbance rates, undisturbed forests will disappear entirely in large tropical humid regions by 2050. Our study suggests that reinforcing actions are needed to prevent the initial degradation that leads to forest clearance in 45% of the cases.

## Teaser

Three decades of satellite observations provide unprecedented information about forest change trajectories in the humid tropics.

## INTRODUCTION

Tropical moist forests (TMF) have immense environmental value. They play an important role in biodiversity conservation, terrestrial carbon cycle, hydrological regimes, indigenous population subsistence, and human health (*1*–*5*). They are increasingly recognized as an essential element of any strategy to mitigate climate change (*6*, *7*). Deforestation and degradation compromise the functioning of tropical forests as an ecosystem, leading to biodiversity loss (*1*, *4*, *5*, *8*, *9*) and reduced carbon storage capacity (*10*–*17*). Deforestation and fragmentation are increasing the risk of viral disease outbreaks (*18*–*20*).

For human well-being, it is a major challenge and shared responsibility to achieve sustainable economic growth and ensure conservation of the remaining TMF. A consistent, accurate, and geographically explicit characterization of long-term disturbances at the pantropical scale is a prerequisite for coherent territorial planning to support the Sustainable Development Goals and the Nationally Determined Contributions of the Paris Agreement (2015). Advances in remote sensing, cloud computing facilities, and free access to the Landsat satellite archive (*21*–*23*) enable the systematic monitoring and consistent dynamic characterization of the entire TMF over a long period. Global maps have been derived to quantify tree cover loss since 2000 (*24*–*25*) and to identify remaining intact forest landscapes (*17*). However, detailed spatial information about the long-term cover changes of TMFs, and, particularly, on forest degradation and postdeforestation recovery, is still lacking. These data are needed to accurately estimate the carbon loss associated with forest disturbances (*2*, *13*, *15*) and to assess their impact on biodiversity (*5*, *8*).

## RESULTS AND DISCUSSION

**Fig. 1:**
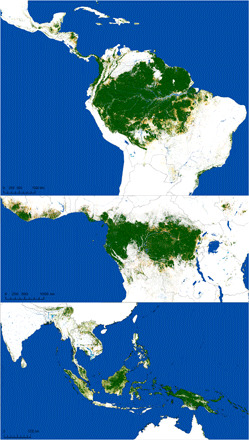
Remaining TMFs. Map of TMFs remaining in January 2020.

**Fig. 2:**
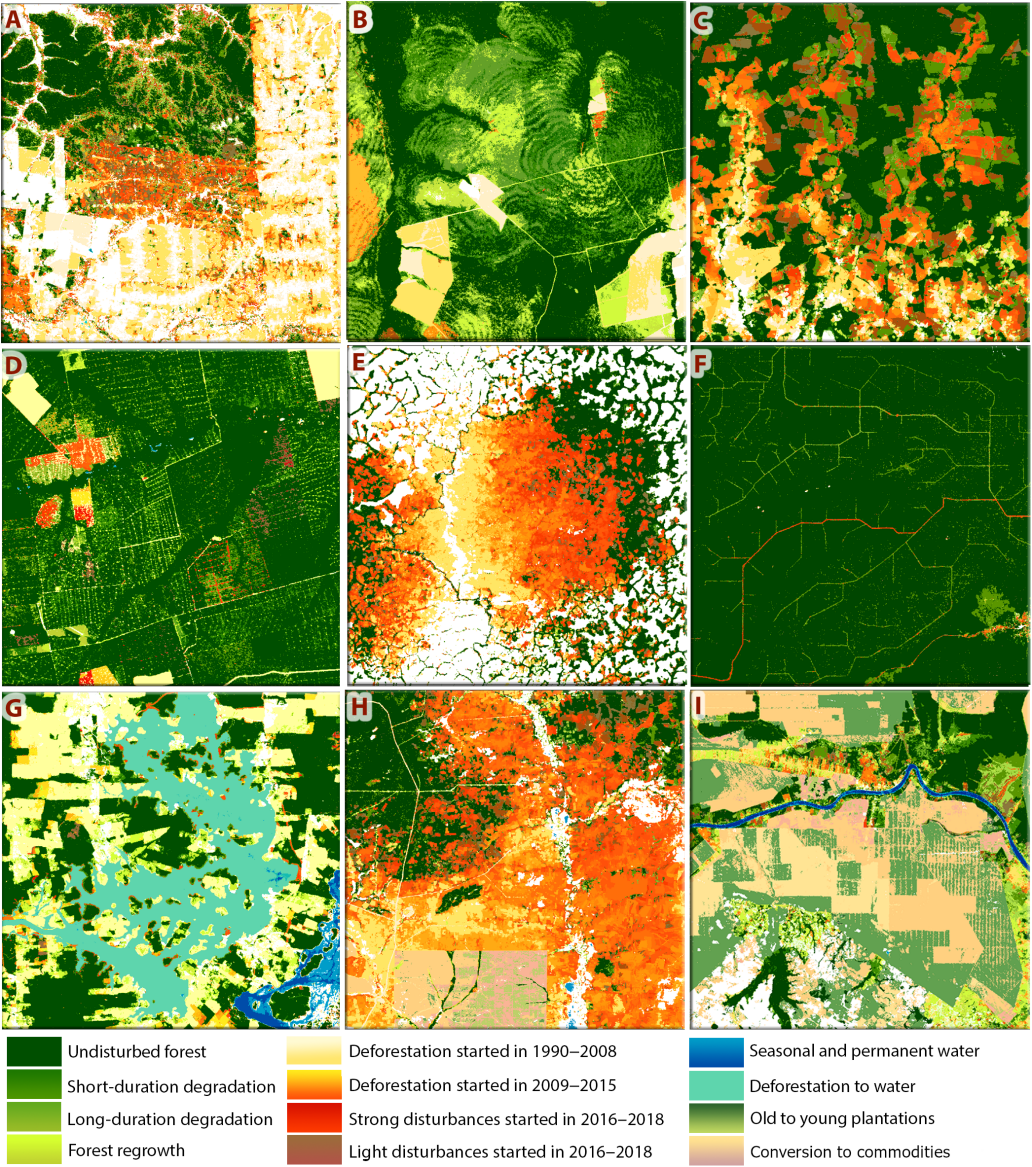
Illustrations of the transition map. Sample patterns of forest cover disturbances (deforestation and degradation) during the period 1990–2019: (**A**) Remaining mangroves and related changes in Guinea-Bissau (14.9°W, 11.1°N), (**B**) fires in the Mato Grosso province of Brazil (53.8°W, 13°S), (**C**) recent deforestation in Colombia (74.4°W, 0.7°N), (**D**) logging in Mato Grosso (54.5°W, 12°S), (**E**) deforestation and degradation caused by the railway in Cameroon (13.4°E, 5.8°N), (**F**) recent selective logging in the Ouésso region of the Republic of Congo (15.7°E, 1.4°N), (**G**) deforestation for the creation of a dam in Malaysia (113.8°E, 2.4°S), (**H**) massive deforestation in Cambodia (105.6°E, 12.7°N), and (**I**) commodities in the Riau province of Indonesia (102°E, 0.4°N). The size of each box is 20 km by 20 km.

Here, we provide unprecedented information through wall-to-wall mapping of TMF cover changes over a long period (January 1990 to December 2019) with Landsat imagery at 0.09-ha resolution (pixel size of 30 m by 30 m), which is freely available from https://forobs.jrc.ec.europa.eu/TMF/ (see Materials and Methods). A description of the forest types included in the TMF is provided in the “Study area and forest types” section. This validated dataset depicts the TMF extent, the related disturbances (natural and anthropogenic degradation or deforestation), and the postdeforestation recovery on an annual basis over the past three decades (see Supplementary Text on annual change dataset, as well as fig. S1). One major innovation is the characterization of the sequential dynamics of changes by providing transition stages from the initial observation period to the end of 2019, i.e., undisturbed forest, degraded forest, forest regrowth, deforested land, conversion to plantations, conversion to water, afforestation, and changes within the mangroves (Figs. 1 and 2 and see Supplementary Text on transition map and figs. S2 to S7), as well as the timing (dates and duration), recurrence, and intensity of each disturbance.

Although no ecosystem may be considered truly undisturbed, as some degree of human impact is present everywhere (*26*), we define an undisturbed TMF as a closed evergreen or semievergreen forest without any disturbance observed over the full Landsat historical dataset (see the “Study area and forest types” section). Consequently, our map baseline of undisturbed forests may include old secondary forests or forests that have been degraded in years before the start of the Landsat archive. This is because we do not have detailed spatial data before the observation period of existing satellite imagery.

At the pantropical scale, the occurrence and extent of forest cover degradation are documented on an annual basis, adding to deforestation data. We define forest degradation as a disturbance in the tree cover canopy that is visible from space over a short time period (less than 2.5 years), leading to a loss of biodiversity and/or carbon storage. The degraded pixel remains forested land (covered by existing or regrowing trees), whereas deforestation leads to the long-term (visible more than 2.5 years) conversion into nonforested land. The detection of the degradation has been achieved through the analysis of each individual valid observation of the Landsat archive (see the “Data” and “Mapping method” sections), making it possible to capture short-duration disturbances, such as selective logging (Fig. 2F and fig. S3), fires (Fig. 2B), and unusual weather events (hurricanes, droughts, and blowdown) (fig. S7).

The accuracy of the disturbance mapping is 91.4%. Uncertainties in the area estimates were quantified on the basis of a sample-based reference in accordance with the latest statistical practices (*27*), indicating an underestimation of the forest disturbance areas by 11.8% (representing 38.4 million ha, with 15 million ha having a confidence interval of 95%) (see the “Validation” section and Supplementary Text on validation, figs. S8 to S10, and tables S1 to S4).

### Main results on degradation

Analysis of the yearly dynamics of TMF disturbances (deforestation or degradation that can have a natural or anthropogenic cause) over the past 30 years underlines the importance of the degradation process in TMF ecosystems, with the following key outcomes (see the “Trend analysis” section, Tables 1 to 10, and fig. S11):

**Table 1:** Areas (in million hectares) of undisturbed TMFs for the years 1990, 1995, 2000, 2005, 2010, 2015, and 2020 (on 1 January) by subregion and continent and relative decline (in percentage) over intervals of 30 years (1990–2019) and 10 years (1990–1999, 2000–2009, and 2010–2019). The values appearing in italic indicate those derived from an average percentage of invalid pixel observations over the baseline TMF domain (Table 9) higher than 40%.

	**Area of undisturbed TMFs on 1 January (Mha)**							**Decline (% of the forest)**			
**Subregion**	**1990**	**1995**	**2000**	**2005**	**2010**	**2015**	**2020**	**[1990–** **2019]**	**[1990–** **1999]**	**[2000–** **2009]**	**[2010–** **2019]**
West Africa	*34.6*	*34.1*	*32.8*	27.4	23.9	20.6	15.6	55.0	*5.0*	27.1	35.0
Central Africa	*223.1*	*221.5*	*216.1*	207.2	201.1	193.7	184.7	17.2	*3.1*	6.9	8.2
Southeast Africa	*15.7*	*15.0*	12.5	10.1	8.9	7.6	6.4	59.2	*20.7*	28.6	27.9
Central America	34.5	32.3	27.4	24.1	21.7	19.6	16.2	53.0	20.8	20.6	25.3
South America	670.6	655.4	628.8	600.9	583.2	568,9	548.2	18.2	6.2	7.3	6.0
Continental Southeast Asia	73.3	67.2	57.7	50.2	44.4	39.9	34.2	53.3	21.2	23.2	22.9
Insular Southeast Asia	237.9	229.5	207.8	192.9	180.8	170.5	159.1	33.1	12.6	13.0	12.0
**Continent**	**1990**	**1995**	**2000**	**2005**	**2010**	**2015**	**2020**	**[1990–** **2019]**	**[1990–** **1999]**	**[2000–** **2009]**	**[2010–** **2019]**
Africa	*273.4*	*270.6*	*261.4*	244.7	234.0	221.9	206.7	24.4	*4.4*	10.5	11.7
Latin America	705.1	687.7	656.1	625.0	604.9	588.5	564.4	19.9	6.9	7.8	6.7
Asia-Oceania	*311.1*	296.7	265.6	243.2	225.2	210.4	193.3	37.9	14.6	15.2	14.2
**Total**	*1289.6*	**1255.0**	**1183.1**	**1112.9**	**1064.0**	**1020.8**	**964.4**	**25.2**	**8.3**	**10.1**	**9.4**

**Table 2:** Areas (in million hectares) of undisturbed and degraded TMFs for the years 1990, 1995, 2000, 2005, 2010, 2015, and 2020 (on 1 January) by subregion and continent and relative decline (in percentage) over intervals of 30 years (1990–2019) and 10 years (1990–1999, 2000–2009, and 2010–2019). The values appearing in italic indicate those derived from an average percentage of invalid pixel observations over the baseline TMF domain (Table 11) higher than 40%.

	**Area of TMFs (undisturbed and degraded) at the end of the year (Mha)**							**Decline (% of the forest)**			
**Subregion**	**1990**	**1995**	**2000**	**2005**	**2010**	**2015**	**2020**	**[1990–** **2019]**	**[1990–** **1999]**	**[2000–** **2009]**	**[2010–** **2019]**
West Africa	*34.6*	*34.3*	*33.3*	29.8	27.4	25.1	22.1	36.0	*3.6*	17.8	19.3
Central Africa	*223.1*	*222.3*	*218.6*	212.7	208.9	204.4	199.9	10.4	*2.0*	4.4	4.3
Southeast Africa	*15.7*	*15.2*	12.9	11.0	10.1	9.2	8.5	45.9	*17.8*	21.8	15.9
Central America	34.5	32.8	29.0	26.8	25.3	24.0	22.1	35.9	16.0	12.8	12.5
South America	670.6	657.7	635.5	613.7	601.0	592.4	581.6	13.3	5.2	5.4	3.2
Continental Southeast Asia	*73.3*	69.1	61.3	55.7	52.0	49.2	46.4	36.6	16.3	15.2	10.6
Insular Southeast Asia	237.9	232.6	218.4	208.9	201.2	194.9	190.2	20.0	8.2	7.9	5.5
**Continent**	**1990**	**1995**	**2000**	**2005**	**2010**	**2015**	**2020**	**[1990–** **2020]**	**[1990–** **2000]**	**[2000–** **2010]**	**[2010–** **2020]**
Africa	*273.4*	*271.7*	*264.8*	253.5	246.4	238.7	230.5	15.7	*3.1*	7.0	6.5
Latin America	705.1	690.5	664.5	640.5	626.3	616.4	603.7	14.4	5.8	5.7	3.6
Asia-Oceania	*311.1*	301.7	279.7	264.5	253.2	244.1	236.7	23.9	10.1	9.5	6.5
**Total**	*1289.6*	**1263.9**	**1209.0**	**1158.5**	**1125.9**	**1099.2**	**1070.9**	**17.0**	**6.2**	**6.9**	**4.9**

**Table 3:** Average annual losses of undisturbed TMF areas (in million hectares) due to deforestation and degradation, from 1990 to 2020 over intervals of 5 years, 30 years (1990–2019), 20 years (2000–2019), and 10 years (1990–1999, 2000–2009, and 2010–2019) by subregion and continent. The values appearing in italic indicate those derived from an average percentage of invalid observations (Table 9) higher than 40%.

	**Annual loss of undisturbed TMF areas (Mha)**									
**Subregion**	**[1990–** **1994]**	**[1995–** **1999]**	**[2000–** **2004]**	**[2005–** **2009]**	**[2010–** **2014]**	**[2015–** **2019]**	**[1990–** **2019]**	**[1990–** **1999]**	**[2000–** **2009]**	**[2010–** **2019]**
West Africa	*0.10*	*0.24*	1.08	0.70	0.67	1.01	0.6	*0.2*	0.9	0.8
Central Africa	*0.33*	*1.07*	1.79	1.22	1.49	1.79	1.3	*0.7*	1.5	1.5
Southeast Africa	*0.13*	0.52	0.47	0.24	0.26	0.24	0.3	0.3	0.4	0.3
Central America	0.45	0.99	0.66	0.47	0.43	0.67	0.6	0.7	0.6	0.6
South America	3.03	5.33	5.56	3.56	2.85	4.14	4.1	4.2	4.6	4.1
Continental Southeast Asia	1.21	1.90	1.50	1.17	0.89	1.14	1.3	1.6	1.3	1.2
Insular Southeast Asia	1.68	4.32	2.98	2.43	2.07	2.28	2.6	3.0	2.7	2.5
**Continent**	**[1990–** **1994]**	**[1995–** **1999]**	**[2000–** **2004]**	**[2005–** **2009]**	**[2010–** **2014]**	**[2015–** **2019]**	**[1990–** **2019]**	**[1990–** **1999]**	**[2000–** **2010]**	**[2010–** **2020]**
Africa	*0.56*	*1.82*	3.34	2.15	2.42	3.04	2.2	*1.2*	2.7	2.6
Latin America	3.48	6.32	6.22	4.03	3.27	4.81	4.7	4.9	5.1	4.8
Asia-Oceania	2.89	6.22	4.48	3.60	2.96	3.42	3.9	4.6	4.0	3.7
**Total**	*6.93*	**14.37**	**14.04**	**9.78**	**8.66**	**11.27**	**10.8**	**10.6**	**11.9**	**11.1**

**Table 4:** Average annual losses of TMF areas (in million hectares) due to deforestation (with or without prior degradation), from 1990 to 2020 over intervals of 5 years, 30 years (1990–2019), 20 years (2000–2019), and 10 years (1990–1999, 2000–2009, and 2010–2019) by subregion and continent. The values appearing in italic indicate those derived from an average percentage of invalid observations (Table 9) higher than 40%.

	**Total deforestation on an annual basis by period (Mha)**									
**Subregion**	**[1990–** **1994]**	**[1995–** **1999]**	**[2000–** **2004]**	**[2005–** **2009]**	**[2010–** **2014]**	**[2015–** **2019]**	**[1990–** **2019]**	**[1990–** **1999]**	**[2000–** **2009]**	**[2010–** **2019]**
West Africa	*0.06*	*0.19*	0.71	0.48	0.45	0.60	0.4	*0.1*	0.6	0.5
Central Africa	*0.17*	*0.74*	1.18	0.76	0.90	0.91	0.8	*0.5*	1.0	0.9
Southeast Africa	*0.10*	0.45	0.38	0.18	0.18	0.14	0.2	0.3	0.3	0.2
Central America	0.34	0.76	0.45	0.29	0.26	0.37	0.4	0.6	0.4	0.3
South America	2.57	4.44	4.35	2.54	1.73	2.16	3.0	3.5	3.4	1.9
Continental Southeast Asia	0.84	1.55	1.13	0.74	0.56	0.54	0.9	1.2	0.9	0.6
Insular Southeast Asia	1.05	2.83	1.91	1.53	1.27	0.94	1.6	1.9	1.7	1.1
**Continent**	**[1990–** **1994]**	**[1995–** **1999]**	**[2000–** **2004]**	**[2005–** **2009]**	**[2010–** **2014]**	**[2015–** **2019]**	**[1990–** **2019]**	**[1990–** **1999]**	**[2000–** **2009]**	**[2010–** **2019]**
Africa	*0.33*	*1.38*	2.26	1.42	1.54	1.65	1.43	0.86	1.84	1.59
Latin America	2.91	5.21	4.80	2.83	1.99	2.53	3.38	4.06	3.82	2.26
Asia-Oceania	1.89	4.38	3.04	2.27	1.83	1.48	2.48	3.14	2.65	1.65
**Total**	*5.14*	**10.97**	**10.10**	**6.52**	**5.35**	**5.66**	**7.29**	**8.06**	**8.31**	**5.51**

**Table 5:** Average annual losses of undisturbed TMF areas (in million hectares) due to degradation (followed or not by deforestation), from 1990 to 2020 over intervals of 5 years, 30 years (1990–2019), 20 years (2000–2019), and 10 years (1990–1999, 2000–2009, and 2010–2019) by subregion and continent. The values appearing in italic indicate those derived from an average percentage of invalid observations (Table 9) higher than 40%.

	**Total degradation on an annual basis by period (Mha)**									
**Subregion**	**[1990–** **1994]**	**[1995–** **1999]**	**[2000–** **2004]**	**[2005–** **2009]**	**[2010–** **2014]**	**[2015–** **2019]**	**[1990–** **2019]**	**[1990–** **1999]**	**[2000–** **2009]**	**[2010–** **2019]**
West Africa	*0.08*	*0.16*	0.87	0.50	0.35	0.61	0.4	*0.1*	0.7	0.5
Central Africa	*0.28*	*0.83*	1.40	0.91	0.92	1.24	0.9	*0.6*	1.2	1.1
Southeast Africa	*0.09*	0.27	0.28	0.14	0.13	0.13	0.2	0.2	0.2	0.1
Central America	0.29	0.64	0.49	0.34	0.27	0.45	0.4	0.5	0.4	0.4
South America	1.25	2.29	2.61	1.83	1.59	2.54	2.0	1.8	2.2	2.1
Continental Southeast Asia	0.88	1.23	1.03	0.81	0.49	0.78	0.9	1.1	0.9	0.6
Insular Southeast Asia	1.16	2.80	1.98	1.39	1.03	1.65	1.7	2.0	1.7	1.3
**Continent**	**[1990–** **1994]**	**[1995–** **1999]**	**[2000–** **2004]**	**[2005–** **2009]**	**[2010–** **2014]**	**[2015–** **2019]**	**[1990–** **2019]**	**[1990–** **1999]**	**[2000–** **2009]**	**[2010–** **2019]**
Africa	*0.45*	*1.26*	2.56	1.55	1.40	1.98	1.53	0.86	2.05	1.69
Latin America	1.54	2.93	3.10	2.16	1.85	2.99	2.43	2.24	2.63	2.42
Asia-Oceania	2.04	4.03	3.00	2.21	1.51	2.43	2.54	3.04	2.61	1.97
**Total**	*4.03*	**8.23**	**8.66**	**5.92**	**4.77**	**7.40**	**6.50**	**6.13**	**7.29**	**6.09**

**Table 6:** Average annual losses of undisturbed TMF areas (in million hectares) due to direct deforestation (without prior degradation), from 1990 to 2020 over intervals of 5 years, 30 years (1990–2019), 20 years (2000–2019), and 10 years (1990–1999, 2000–2009, and 2010–2019) by subregion and continent. The values appearing in italic indicate those derived from an average percentage of invalid observations (Table 9) higher than 40%.

	**Annual direct deforestation by period (Mha)**									
**Subregion**	**[1990–** **1994]**	**[1995–** **1999]**	**[2000–** **2004]**	**[2005–** **2009]**	**[2010–** **2014]**	**[2015–** **2019]**	**[1990–** **2019]**	**[1990–** **1999]**	**[2000–** **2009]**	**[2010–** **2019]**
West Africa	*0.02*	*0.08*	0.21	0.19	0.32	0.40	0.2	*0.0*	0.2	0.04
Central Africa	*0.05*	*0.24*	0.38	0.31	0.57	0.56	0.4	*0.1*	0.3	0.6
Southeast Africa	*0.05*	0.24	0.19	0.10	0.13	0.10	0.1	0.1	0.1	0.1
Central America	0.16	0.34	0.17	0.13	0.16	0.22	0.2	0.2	0.2	0.2
South America	1.78	3.05	2.95	1.73	1.26	1.61	2.1	2.4	2.3	1.4
Continental Southeast Asia	0.33	0.66	0.48	0.36	0.41	0.36	0.4	0.5	0.4	0.4
Insular Southeast Asia	0.52	1.53	1.00	1.03	1.04	0.62	1.0	1.0	1.0	0.8
**Continent**	**[1990–** **1994]**	**[1995–** **1999]**	**[2000–** **2004]**	**[2005–** **2009]**	**[2010–** **2014]**	**[2015–** **2019]**	**[1990–** **2019]**	**[1990–** **1999]**	**[2000–** **2009]**	**[2010–** **2019]**
Africa	*0.11*	*0.56*	0.78	0.60	1.02	1.06	0.69	0.34	0.69	1.04
Latin America	1.94	3.39	3.12	1.86	1.42	1.82	2.26	2.66	2.49	1.62
Asia-Oceania	0.85	2.19	1.48	1.39	1.45	0.99	1.39	1.52	1.44	1.22
**Total**	*2.90*	**6.14**	**5.38**	**3.86**	**3.89**	**3.87**	**4.34**	**4.52**	**4.62**	**3.88**

**Table 7:** Average annual losses of undisturbed TMF areas (in million hectares) due to annual degradation before deforestation, from 1990 to 2020 over intervals of 5 years, 30 years (1990–2019), 20 years (2000–2019), and 10 years (1990–1999, 2000–2009, and 2010–2019) by subregion and continent. The values appearing in italic indicate those derived from an average percentage of invalid observations (Table 9) higher than 40%.

	**Annual degradation before deforestation by period (Mha)**									
**Subregion**	**[1990–** **1994]**	**[1995–** **1999]**	**[2000–** **2004]**	**[2005–** **2009]**	**[2010–** **2014]**	**[2015–** **2019]**	**[1990–** **2019]**	**[1990–** **1999]**	**[2000–** **2009]**	**[2010–** **2019]**
West Africa	*0.04*	*0.11*	0.50	0.28	0.14	0.20	0.2	*0.1*	0.4	0.2
Central Africa	*0.12*	*0.50*	0.79	0.45	0.33	0.35	0.4	*0.3*	0.6	0.3
Southeast Africa	*0.06*	0.21	0.19	0.08	0.05	0.04	0.1	0.1	0.1	0.0
Central America	0.18	0.42	0.28	0.16	0.10	0.16	0.2	0.3	0.2	0.1
South America	0.79	1.40	1.40	0.81	0.47	0.55	0.9	1.1	1.1	0.5
Continental Southeast Asia	0.51	0.89	0.65	0.38	0.15	0.18	0.5	0.7	0.5	0.2
Insular Southeast Asia	0.53	1.31	0.91	0.50	0.23	0.31	0.6	0.9	0.7	0.3
**Continent**	**[1990–** **1994]**	**[1995–** **1999]**	**[2000–** **2004]**	**[2005–** **2009]**	**[2010–** **2014]**	**[2015–** **2019]**	**[1990–** **2019]**	**[1990–** **1999]**	**[2000–** **2009]**	**[2010–** **2019]**
Africa	*0.22*	*0.82*	1.48	0.82	0.52	0.59	0.74	0.52	1.15	0.55
Latin America	0.98	1.82	1.68	0.97	0.57	0.71	1.12	1.40	1.33	064
Asia-Oceania	1.05	2.19	1.56	0.88	0.38	0.49	1.09	1.62	1.22	0.44
**Total**	*2.24*	**4.83**	**4.72**	**2.66**	**1.74**	**1.79**	**2.95**	**3.54**	**3.69**	**1.63**

**Table 8:** Average annual losses of TMF areas (in million hectares) due to deforestation followed by regrowth, from 1990 to 2020 over intervals of 5 years, 30 years (1990–2019), 20 years (2000–2019), and 10 years (1990–1999, 2000–2009, and 2010–2019) by subregion and continent. The values appearing in italic indicate those derived from an average percentage of invalid observations (Table 9) higher than 40%.

	**Total deforestation followed by regrowth on an annual basis by period (Mha)**									
**Subregion**	**[1990–** **1994]**	**[1995–** **1999]**	**[2000–** **2004]**	**[2005–** **2009]**	**[2010–** **2014]**	**[2015–** **2019]**	**[1990–** **2019]**	**[1990–** **1999]**	**[2000–** **2009]**	**[2010–** **2019]**
West Africa	*0.00*	*0.00*	0.01	0.04	0.06	0.03	0.0	*0.0*	0.0	0.0
Central Africa	*0.02*	*0.04*	0.07	0.10	0.13	0.06	0.1	*0.0*	0.1	0.1
Southeast Africa	*0.00*	0.01	0.02	0.03	0.03	0.01	0.0	0.0	0.0	0.0
Central America	0.05	0.09	0.07	0.07	0.05	0.02	0.1	0.1	0.1	0.0
South America	0.21	0.40	0.50	0.49	0.37	0.20	0.4	0.3	0.5	0.3
Continental Southeast Asia	0.10	0.20	0.24	0.23	0.14	0.06	0.2	0.2	0.2	0.1
Insular Southeast Asia	0.11	0.33	0.44	0.42	0.30	0.15	0.3	0.2	0.4	0.2
**Continent**	**[1990–** **1994]**	**[1995–** **1999]**	**[2000–** **2004]**	**[2005–** **2009]**	**[2010–** **2014]**	**[2015–** **2019]**	**[1990–** **2019]**	**[1990–** **1999]**	**[2000–** **2009]**	**[2010–** **2019]**
Africa	*0.02*	*0.06*	0.10	0.17	0.21	0.10	0.11	0.04	0.13	0.15
Latin America	0.26	0.48	0.58	0.56	0.43	0.22	0.42	0.37	0.57	0.32
Asia-Oceania	0.21	0.53	0.68	0.65	0.44	0.21	0.45	0.37	0.66	0.32
**Total**	*0.50*	**1.06**	**1.36**	**1.37**	**1.08**	**0.53**	**0.98**	**0.78**	**1.37**	**0.80**

**Table 9:** Average percentage of invalid observations over the baseline TMF domain. Average percentage of invalid observations over the baseline TMF domain (A) per year from 1982 to 2020 and (B) over intervals of 5 years (except for the first interval that includes 8 years), by subregion and continent. The values appearing in italic indicate an average percentage of invalid observations higher than 40%.

**A**								
	**Average % of invalid observations (over the total forest domain, per year)**							
**Subregion**	**1982**	**1990**	**1995**	**2000**	**2005**	**2010**	**2015**	**2019**
West Africa	*100.0*	*90.8*	*84.3*	*71.8*	5.9	0.7	0.2	0.0
Central Africa	*100.0*	*97.7*	*87.6*	*67.8*	10.2	3.7	1.9	0.0
Southeast Africa	*100.0*	*92.1*	*41.5*	8.4	1.5	1.0	0.5	0.0
Central America	*69.4*	17.0	5.8	1.8	0.7	0.2	0.0	0.0
South America	*55.6*	1.7	0.8	0.3	0.1	0.0	0.0	0.0
Continental Southeast Asia	*55.2*	*49.1*	2.0	1.0	0.0	0.0	0.0	0.0
Insular Southeast Asia	34.6	31.2	6.5	1.9	0.2	0.1	0.0	0.0
**Continent**	**1982**	**1990**	**1995**	**2000**	**2005**	**2010**	**2015**	**2019**
Africa	*100.0*	*93.5*	*71.2*	*49.3*	5.9	1.8	0.9	0.0
Latin America	*62.5*	9.4	3.3	1.0	0.4	0.1	0.0	0.0
Asia-Oceania	*44.9*	*40.2*	4.3	1.4	0.1	0.1	0.0	0.0
**Total**	69.1	47.7	**26.2**	**17.3**	**2.1**	**0.7**	**0.3**	**0.0**
**B**								
	**Average % of invalid observations (over the total forest domain, per year)**							
**Subregion**	**[1982–1989]**	**[1990–1994]**		**[1995–1999]**	**[2000–2004]**	**[2005–2009]**	**[2010–2014]**	**[2015–2019]**
West Africa	*98.1*	*87.1*		*82.5*	40.0	2.8	0.4	0.0
Central Africa	*99.4*	*94.7*		*80.8*	37.2	6.7	2.9	0.4
Southeast Africa	*97.9*	*80.8*		20.7	3.9	1.3	0.8	0.1
Central America	*50.1*	12.4		4.3	1.2	0.4	0.1	0.0
South America	30.5	1.2		0.6	0.2	0.0	0.0	0.0
Continental Southeast Asia	*54.5*	14.6		1.3	0.4	0.0	0.0	0.0
Insular Southeast Asia	34.3	16.3		4.2	0.8	0.1	0.1	0.0
**Continent**	**[1982–1989]**	**[1990–1994]**		**[1995–1999]**	**[2000–2004]**	**[2005–2009]**	**[2010–2015]**	**[2015–2020]**
Africa	*99.1*	*92.9*		*77.6*	35.7	5.9	2.4	0.3
Latin America	31.3	1.6		0.8	0.2	0.1	0.0	0.0
Asia-Oceania	29.5	12.0		3.7	1.3	0.3	0.2	0.0
**Total**	**21.2**	31.9		**23.1**	**10.1**	**2.3**	**0.8**	**0.1**

**Table 10:** Total areas and proportions of TMF disturbances (deforestation without regrowth, regrowth after deforestation, and forest degradation) and reforestation areas (initially other land cover) over the period 1990–2020 for each subregion and continent (areas in million hectares and proportions in percentages). LC, land cover.

	**Disturbed areas (Mha)**				**% of total disturbances**			**% of undisturbed forest in 1990**				
**Subregion**	**Deforestation**	**Regrowth**	**Degradation**	**Total**	**Deforestation**	**Regrowth**	**Degradation**	**Deforestation**	**Regrowth**	**Degradation**	**Total**	**Reforestation** **(from other LC)**
West Africa	11.8	0.7	6.5	19.0	61.9	3.7	34.5	34.0	2.0	18.9	55.0	0.4
Central Africa	21.2	2.1	15.1	38.4	55.2	5.4	39.4	9.5	0.9	6.8	17.2	1.1
Southeast Africa	6.7	0.5	2.1	9.3	71.9	5.7	22.4	42.5	3.4	13.3	59.2	0.2
Central America	10.6	1.8	5.9	18.3	58.0	9.7	32.3	30.7	5.1	17.1	53.0	0.7
South America	78.1	10.9	33.4	122.4	63.8	8.9	27.3	11.6	1.6	5.0	18.2	4.0
Continental Southeast Asia	22.0	4.9	12.2	39.1	56.2	12.4	31.4	30.0	6.6	16.7	53.3	2.0
Insular Southeast Asia	38.9	8.7	31.1	78.8	49.4	11.1	39.5	16.4	3.7	13.1	33.1	1.6
**Continent**	**Deforestation**	**Regrowth**	**Degradation**	**Total**	**Deforestation**	**Regrowth**	**Degradation**	**Deforestation**	**Regrowth**	**Degradation**	**Total**	**Reforestation** **(from other LC)**
Africa	39.6	3.3	23.8	66.7	59.4	4.9	35.6	14.5	1.2	8.7	24.4	1.6
Latin America	88.7	12.6	39.3	140.7	63.1	9.0	27.9	12.6	1.8	5.6	19.9	4.7
Asia-Oceania	60.9	13.6	43.4	117.8	51.7	11.5	36.8	19.6	4.4	13.9	37.9	3.6
**Total**	189.2	29.5	106.5	325.2	58.2	9.1	32.7	14.9	2.3	8.4	25.7	10.0

**Fig. 3:**
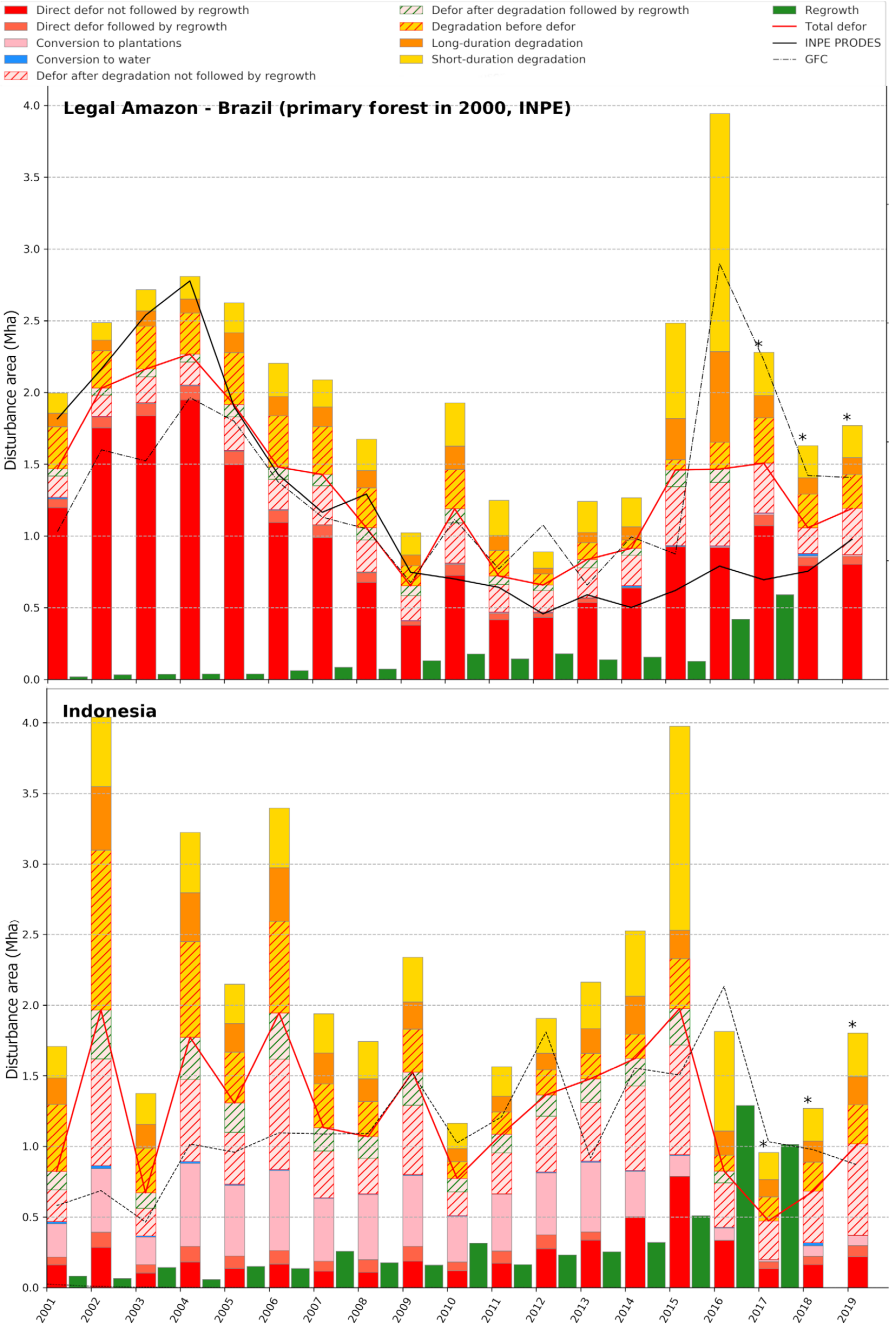
Dynamics of annual disturbed areas for the Amazônia Legal region and Indonesia. Dynamics of annual disturbed areas from 2001 to 2019 for the Amazônia Legal region of Brazil within the primary forest extent in 2000 from National Institute for Space Research (INPE) and Indonesia using the entire TMF extent (undisturbed and degraded) in 2000 (*x* axis in years and *y* axis in million hectares) in comparison with GFC loss and the PRODES data for the Amazônia Legal region of Brazil. Asterisks (*) indicate that the average proportion of disturbance types within total disturbances over the period 2005–2014 is used to distribute the disturbance types for years 2017 to 2019. Defor, deforestation.

**Fig. 4:**
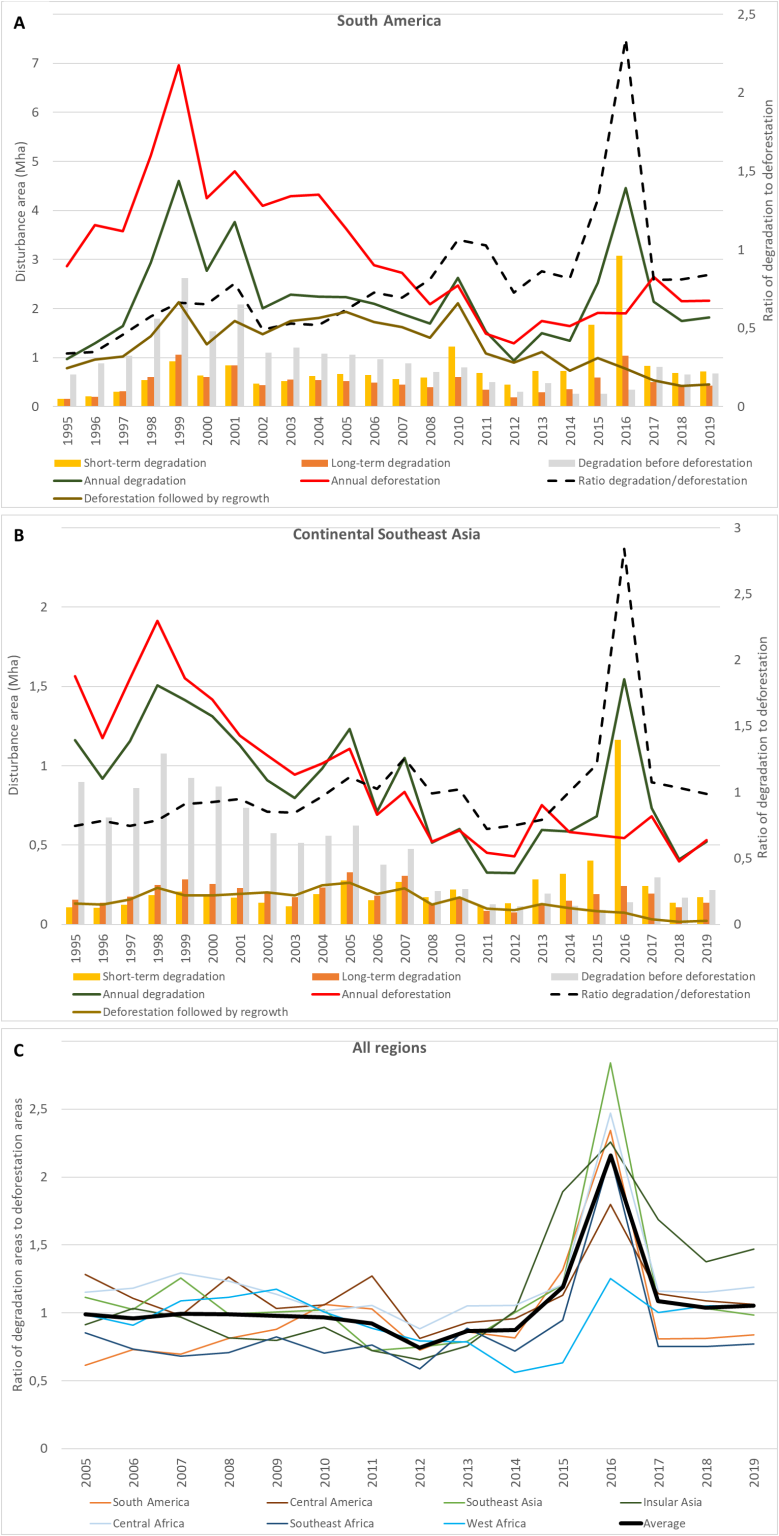
Evolution of annual deforestation and degradation at the regional scale. Evolution of annual deforestation and degradation (**A**) over the past 25 years in South America, (**B**) in continental Southeast Asia, and (**C**) over the past 15 years in all regions. “Short-term degradation” refers to disturbances that have been observed over less than 1 year, mostly due to selective logging, natural events, and light-impact fires, whereas “long-term degradation” refers mostly to fires that are detected over a period of 1 to 2.5 years.

1) Over the past three decades, 218.7 million ha of TMF has disappeared, and 106.5 million ha is in a degraded state (Tables 2 and 10). This represents 10% of the 1070.9 million ha of forest area remaining in January 2020. Degraded forests account for 33% of the observed changes in forest cover (i.e., from total changes including deforested land and forest regrowth) with high variability between regions and countries, ranging from 96% in Venezuela, 74% in Gabon, and 69% in Papua New Guinea through to 21% in Brazil and Madagascar, and 13% in Cambodia (table S6). As much as 40.7% of the degraded forests are in Asia-Oceania (compared with 36.9% in Latin America and 22.3% in Africa) (Table 10).

2) About 84.5% of the degraded forests (i.e., 90 million ha) is attributable to short-term disturbances (observed for less than 1 year and mostly due to selective logging, natural events, and light-impact fires), of which 30 million ha has been degraded two or three times over the past 30 years (observed each time over a short period). The remaining 15.5% (16.5 million ha) is mainly the result of intense fires, with a disturbance duration (period in which the disturbance effect is visible on Landsat imagery) of 1 to 2.5 years.

3) As much as 45.4% of the degradation (88.6 million ha) is a precursor of deforestation events occurring, on average, after 7.5 years (without substantial variability between continents). This is particularly true for Southeast Africa and Southeast Asia, which show, respectively, 60.4% (with 65% for Madagascar) and 53% (with 59% for Cambodia) of degraded forests becoming deforested after a recovery period (Table 7). These proportions are underestimated because 45.4% of recent degradation (e.g., in the past 7 years) will most likely lead to deforestation in future years.

4) A further 30.3% of the undisturbed forest areas (291.8 million ha) is potentially disturbance edge-affected forests, i.e., located within 120 m of a disturbance (see Materials and Methods). This proportion indicates greater forest fragmentation in Asia (45.2%) compared with other continents (25.6 and 28.9% in the Americas and Africa, respectively).

5) As much as 82.8% of the TMF mapped as degraded in December 2019 corresponds to short-term disturbances that have never been identified at the pantropical scale. Over the period covered by the Global Forest Change (GFC) product (*24*), i.e., 2001 to 2019, about 21.2 million ha has been captured as tree cover loss compared with 86 million ha detected as degraded forests by our study during the same period (see the “Comparison with the GFC dataset” section, Fig. 3, and table S5).

6) We show that the annual rate of degradation is closely related to climatic conditions (Figs. 3 and 4 and fig. S11). Whereas the trends in deforestation rates seem to be related to changes in national territorial policies, degradation rates usually show peaks during drought periods and do not seem to be affected by forest conservation policies. The drought conditions that occurred during strong and very strong El Niño–Southern Oscillation (ENSO) events of 1997–1998 and 2015–2016 were optimal for forest fires (*28*–*30*) and resulted in a significant increase in forest degradation (*29*). The impact of these fires in 2015–2016 is particularly strong and visible in all regions except Southeast Africa.

Our results stress the paramount importance of (i) integrating measures for reducing degradation in forest conservation and climate mitigation programs and (ii) considering forest degradation as a risk factor of deforestation and as an indicator of climate change and climate oscillations. We expect that improved knowledge of forest degradation processes and the resulting fragmentation will help to make accurate assessments of the anthropogenic impact on tropical ecosystem services and of the effects on biosphere-atmosphere-hydrosphere feedbacks. Future policies will have to account for this finding.

### Main results on deforestation and postdeforestation regrowth

Deforestation in TMF cover is documented in an unprecedentedly comprehensive manner: (i) by covering a 30-year period of analysis, (ii) by separating direct deforestation of undisturbed forest from deforestation of degraded forest, (iii) by mapping postdeforestation recovery (or forest regrowth), (iv) by identifying specific forest conversion to commodities or water (Fig. 2, G and I), (v) by including changes within the mangroves (Fig. 2A), and (vi) by documenting each deforestation event at the pixel level by its timing (date and duration), intensity, recurrence, and, when appropriate, start date and duration of postdeforestation regrowth. Direct deforestation is characterized by the full removal of trees within a few months, while deforestation of degraded forest is characterized by the removal of remaining and regrowing trees after a partial removal that happened at least 1 year before.

Overall, 17.2% of the initial TMF area (i.e., 218.7 million ha of 1289.6 million ha) has disappeared since 1990, falling to 1070.9 million ha of TMF in January 2020 (Table 4). We report the gross loss of TMF area for the entire pantropical region varying from 5.5 million to 8.3 million ha per year within the period (Table 11). A comparison with previous studies results in the following outcomes:

**Table 11:** Comparison of estimates of annual deforested areas (in million hectares per year) from previous studies and our study, over the tropical belt, over the three continents and Brazil.

**Source**		**GFC (*24*)**		**Sample-** **based (*32*)**	**FAO national** **statistics (*31*)**	**PRODES-** **INPE (*30*)**	**This study**		
**Forest extent**		**Whole TMF** **(undisturbed** **and degraded)**	**Primary** **forest from** **INPE**	**Natural** forest*	**All tropical** **forests** **(evergreen and** **deciduous)**	**Primary** **forest**	**Whole TMF**	**TMF** **excluding** **the tree** **plantations**	**Primary** **forest from** **INPE**
**Pantropical** **region**	2001–2010	4.67			7.24		7.72		
	2001–2012	4.80		6.5 ± 0.7			7.19	6.44	
	2001–2015	5.07			6.66		6.95		
	2001–2019	5.79					6.66		
**Africa**	2001–2012	0.73		1.21 ± 0.4			1.60	1.57	
	2001–2019	1.28					1.64		
**Latin America**	2001–2012	2.19		3.7 ± 0.5			3.25	3.19	
	2001–2019	2.41					2.93		
**Asia-Oceania**	2001–2012	1.89		1.6 ± 0.4			2.34	1.67	
	2001–2019	2.10					2.09		
**Brazil**	2001–2010	1.61	1.35			1.65	2.55		1.57
	2001–2012	1.54	1.26	2.1 ± 0.3		1.47	2.32	2.27	1.42
	2010–2019	1.64	1.34			0.67	1.63		1.04
	2001–2019	1.64	1.35			1.19	2.10		1.31

*Evergreen and deciduous, excluding manage forests (e.g., tree plantations).

1) Estimations reported by Food and Agriculture Organization of the United Nations (FAO) national statistics (*31*) and the sample-based estimations from Tyukavina *et al*. (*32*) for the natural tropical forest, including both moist and dry forest types, are higher by 0.9 and 27%, respectively, compared with our TMF deforestation rates (which exclude the conversion to tree plantations to approach the “natural forest” definition of these two studies) for the same period (Table 11). At the continental scale, Tyukavina *et al*. (*32*) shows lower estimates than our study for Africa (−23%) and for Asia (−4%) and higher estimates for Latin America (+16%).

2) Comparison with GFC loss (see the “Comparison with the GFC dataset” section and Fig. 3) (*24*) shows a lower deforestation rate (−33%) than our study for the period 2000–2012 over the same forest extent (using our TMF extent for the year 2000) (Table 11). Underestimation of GFC loss has been documented by previous studies (*32*, *33*). Tyukavina *et al*. (*32*) reported an underestimation of GFC loss of 19.4% when considering the entire forest cover (moist and deciduous) loss during the period 2001–2012, with a larger underestimation for Africa (−39.4%) than for other continents (−13% for Latin America and −5.7% for Asia). The ranking of this underestimation by continent is consistent with the ranking observed in our study (first, Africa; second, Latin America; and, third, Asia). The differences with GFC loss are explained by three specific assets of our approach: (i) the use of single-date images, enabling the detection of short-duration disturbance events (i.e., visible from space for only a few weeks), compared with the use of annual syntheses; (ii) a dedicated algorithm for TMF, enabling the monitoring of seven classes of forest cover change, compared with the global monitoring of forest clearance; and (iii) cloud masking and quality control optimized for equatorial regions, enabling a more comprehensive analysis of the Landsat archive.

3) Comparison with the Brazilian Projeto de Monitoramento do Desmatamento na Amazônia Legal por Satélite (PRODES) data (*30*) using their primary forest extent (Fig. 3) shows a similar decrease in annual deforestation rates between the 2000s and the past decade; this can be related to a set of economic and public policy actions (*29*). Differences in the deforestation rates are observed (i) during the period 2001–2004 with a higher deforestation rate for PRODES (2.32 million ha/year) compared with our study (2 million ha/year) and with GFC loss (1.53 million ha/year), and (ii) in the past 10 years, there has been a lower average deforestation rate for PRODES compared with our study and GFC loss (0.67 million, 1.1 million, and 1.34 million ha per year, respectively) (Table 11). These differences are accentuated in the past 5 years (0.77 million, 1.33 million, and 1.76 million ha per year, respectively). Discrepancies in area estimates between our product and the PRODES data are explained by (i) difference in minimum mapping units (0.09 ha compared with 6.25 ha in PRODES) and (ii) the impacts of strong fires that are captured in our study (deforestation followed by forest regrowth) and in GFC loss but are discarded in the PRODES approach (because they are not considered deforestation).

This study documents, in an unprecedented manner, the extent and age of young secondary forests for the entire pantropical domain. These secondary forests are defined here as tree cover regrowth (visible for at least 3 years) after a full removal of tree cover that has remained without regrowing trees for at least 2.5 years. They grow rapidly under tropical moist conditions and absorb large amounts of carbon, but they were poorly documented. We show that 13.5% of the deforested areas (i.e., 29.5 million ha) is recovering in a subsequent stage, with 33% of these secondary forests aged more than 10 years at the end of 2019 (Table 10). The proportion of secondary forests within total deforestation is higher in Asia (18.3%) than in Latin America (12.3%) and Africa (7.9%). The disturbance events followed by forest regrowth include intense fires, which are accentuated by drought conditions. This is very visible for South America (Fig. 4) for the years 1997–1998 and 2010. In addition, 10 million ha is characterized as evergreen vegetation regrowth of areas initially classified as nonforest cover, i.e., which can be considered as forestation (i.e., afforestation and reforestation) aged more than 10 years.

This study confirms that most of the deforestation caused by the expansion of oil palm and rubber and assigned to the commodity classes in our study (Figs. 2I and 4B and see Supplementary Text on ancillary datasets, fig. S11, and table S6), is concentrated in Asia with 18.3 million ha (representing 86% of the entire TMF conversion to plantations) and more specifically in Indonesia (57.4%) and Malaysia (23.8%).

### Deforestation and degradation trends

**Fig. 5:**
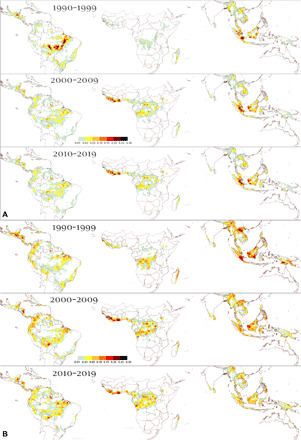
Hot spots of deforestation and degradation. Evolution of total deforested (**A**) and degraded (**B**) areas (per box of 1° latitude by 1° longitude size; scale in million hectares) within the labeled time intervals (1990–1999, 2000–2009, and 2010–2019).

The evolution of deforestation and degradation over the past three decades shows the highest peaks of annual disturbance in Latin America and Southeast Asia during the period 1995–2000, with 6.3 million and 6.2 million ha/year, respectively. The ENSO of 1997–1998 may, at least partially, explain these peaks of forest disturbance, in particular, for Indonesia and Brazil, where these peaks manifest themselves in the annual change trends, with the highest proportion of degradation events over the total disturbance areas (Figs. 4 and 5 and fig. S11). From 2000 to 2004 and from 2015 to 2019, the disturbance rates declined by half in South America and by 45% in Southeast Africa and continental Southeast Asia. Brazil, which accounts for 29% of the world’s remaining TMF, made a large contribution to this reduction (from 4.3 million ha/year down to 2.1 million ha/year) (Figs. 3, 4, and 5, Table 11, table S6, and fig. S11).

In recent years, our study shows a marked increase in disturbance rates (deforestation and degradation) (+2.1 million ha/year for the past 5 years compared with the period 2005–2014), reaching a level close to that of the early 2000s and with the highest increases observed in West Africa and Latin America (48% higher) (Table 3). Degradation is the main contributor of this recent increase (average increase of 38% while annual deforestation declined by 5%) (Tables 5 and 7), notably caused by specific climatic conditions in 2015–2016 (Figs. 4 and 5) (*30*). The Asia-Oceania region shows a smaller increase in degradation rate (31%) than Africa (34%) and Latin America (49%) and a much bigger decline in deforestation rate (28%) compared with Africa (5%) and Latin America (12%) (Tables 4 to 8).

### Undisturbed TMF decline and projections

Since 1990, the extent of undisturbed TMF has shrunk by 23.9% with an average loss rate of 10.8 million ha/year (Tables 1 and 3). The decline of undisturbed TMF is particularly marked for Ivory Coast (81.5% of their extent in 1990), Mexico (73.7%), Ghana (70.8%), Madagascar (69%), Vietnam (67.8%), Angola (67.1%), Nicaragua (65.8%), Lao People’s Democratic Republic (PDR) (65.1%), and India (63.9%) (table S6). If the average rates of the period 2010–2019 remain constant in the near to medium term (see Materials and Methods and fig. S12), then undisturbed TMF would disappear by 2026–2029 in Ivory Coast and Ghana; by 2040 in Central America and Cambodia; by 2050 in Nigeria, Lao PDR, Madagascar, and Angola; and by 2065 for all the countries of continental Southeast Asia and Malaysia. By 2050, a total of 15 countries, including Malaysia (the country with the ninth biggest TMF), will lose more than 50% of their undisturbed forests (table S6).

It is now possible to monitor deforestation and degradation in TMFs consistently over a long historical period and at fine spatial resolution. The mapping of forest transition stages will make it possible to derive more targeted indicators to measure the achievements in forest, biodiversity, health, and climate policy goals from local to international levels (*34*). Our study shows that TMFs are disappearing at much faster rates than previously estimated, underlining the precursor role of forest degradation in this process. These results should make decision makers aware of the pressing need to reinforce actions for preserving tropical forests, in particular, by avoiding the initial degradation that most likely leads to subsequent forest clearance.

## MATERIALS AND METHODS

### Study area and forest types

Our study covers the TMFs, which include all closed forests in the humid tropics (*35*) with two main forest types (*36*): the tropical rain forest and the tropical moist deciduous forest. Tropical rain forest is found in permanently humid areas, i.e., those with, at most, only limited seasonality in rainfall distribution, while tropical moist deciduous forest, also called monsoon forest, is found in areas with a distinct dry season. Each of these two main forest types can be divided into numerous subtypes (*37*), such as the lowland evergreen moist forest, the montane moist forest, the mangrove forest, the swamp forest, and the tropical semievergreen moist forest. “Evergreenness” varies from permanently evergreen to evergreen seasonal (mostly evergreen but with individual trees that may lose their leaves), semievergreen seasonal (up to about one-third of the top canopy can be deciduous, although not necessarily leafless at the same time), and moist deciduous (dominant deciduous species with evergreen secondary canopy layer). The TMFs are characterized by low variability in annual temperature and high levels of rainfall (>200 cm annually). In the Holdridge life zones classification scheme (*38*), the TMFs include the moist forest, the wet forest, and the rain forest. Our study area covers the following Global Ecological Zones (*39*): “tropical rain forest,” “tropical moist forest,” “tropical mountain system,” and “tropical dry forest” (fig. S13), stopping at the borders of China, Pakistan, Uruguay, and United States. The TMFs are located mostly in the tropical moist and humid climatic domains, but they also include small areas of gallery forests in the tropical dry domain.

We do not intend to map specifically intact or primary forest, as the Landsat observation period is too short to discriminate between never-cut primary forest and secondary forest older than the observation period. However, by documenting all the disturbances observed over the past three decades, the remaining undisturbed TMF in 2019 is getting closer to the primary forest extent. While our entire TMF, including undisturbed and degraded forests, in 1990 and 2019 are comparable, our undisturbed forest of 1990 and 2019 are less comparable.

### Data

The Landsat archive is the only free and long-term satellite image record suitable for analyzing land cover changes on an annual basis over a long period at fine spatial resolution. We used the entire L1T archive (orthorectified top of atmosphere reflectance) acquired from July 1982 to December 2019 from the following Landsat sensors: Thematic Mapper (TM) on board Landsat 4 and Landsat 5, Enhanced Thematic Mapper Plus (ETM+) on board Landsat 7, and the Operational Land Imager on board Landsat 8 (*23*, *40*–*42*). Landsat 4 was launched in July 1982 and collected images from its TM sensor until December 1993. Landsat 5 was launched in March 1984 and collected images until November 2011. Landsat 7 was launched in April 1999 and acquired images normally until May 2003 when the scan line corrector (SLC) failed (*43*). All Landsat 7 data acquired after the date of the SLC failure have been used in our analysis. Landsat 8 began operational imaging in April 2013.

The Landsat archive coverage presents large geographical and temporal unevenness (*40*, *44*). The main reason for the limited availability of images for some regions is that Landsat 4 and Landsat 5 had no onboard data recorders, and links with data relay satellites failed over time; cover was therefore often limited to the line of sight of receiving stations (*42*). Commercial management of the program from 1985 to the early 1990s led to data being acquired mostly when preordered (*40*). From 1999 onward, the launch of Landsat 7 and its onboard data recording capabilities, associated with the continuation of the Landsat 5 acquisitions, considerably improved global coverage.

In the tropical regions, Africa is particularly affected by the limited availability of image acquisitions, especially in the first part of the archive. From a total of around 1,370,860 Landsat scenes that were available for our study area, only 265,098 were located in Africa (compared with 573,589 and 532,173 for Latin America and Asia, respectively). The most critical area is located around the Gulf of Guinea, with an overall average number of valid observations (i.e., without clouds, hazes, sensor artifacts, and geolocation issues) over the full archive (fig. S14) amounting to less than 50 per location (pixel) and with the first valid observations starting mostly at the end of the 1990s (fig. S15). Small parts of Ecuador, Colombia, the Solomon Islands, and Papua New Guinea present a similarly low number of total valid observations, often with an earlier first valid observation around the end of the 1980s. Apart from these regions, the first valid observation occurs mostly within the periods 1982–1984, 1984–1986, or 1986–1988 for Latin America, Africa, and Southeast Asia, respectively.

The average number of annual valid observations (fig. S16) shows a gradual increase during the 38-year period for the three continents, with two major jumps: in 1999 with the launch of Landsat 7 and in 2013 with the launch of Landsat 8. There is also a clear drop in 2012 for Southeast Asia and Latin America with the decommissioning of Landsat 5 in November 2011 and a small drop in 2003 as a consequence of the Landsat 7 SLC-off issue. There are major differences between Africa and the two other continents: Africa has significantly fewer valid observations, in particular, during the period 1982–1999, and a much larger increase in number of observations from 2013.

The geographical unevenness of the first year of acquisition constrains the monitoring capability period. Our method accounts for this constraint notably by recording the effective duration of the archive at the pixel level (see next subsection). Data quality issues affecting the Landsat collection were addressed by excluding pixels where (i) detector artifacts occur (manifested as random speckle or striping), (ii) one or more spectral bands are missing (typically occurring at image edges), or (iii) scene geolocation is inaccurate.

### Mapping method

To map the extent and changes of the TMF over a long period, we developed an expert system that exploits the multispectral and multitemporal attributes of the Landsat archive to identify the main change trajectories over the past three decades and that uses ancillary information to identify subclasses of forest conversion (see Supplementary Text on ancillary data). The inference engine of our system is a procedural sequential decision tree, where the expert knowledge is represented in the form of rules. Techniques for big data exploration and information extraction, namely, visual analytics (*45*) and evidential reasoning (*46*), were used similarly to a recent study dedicated to global surface water mapping (*44*). The advantages of these techniques for remotely sensed data analysis are presented in this previous study (*44*). These notably include accounting for uncertainty in data, guiding and informing the expert’s decisions, and incorporating image interpretation expertise and multiple data sources. The expert system was developed and operated in the Google Earth Engine (GEE) geospatial cloud computing platform (*22*). The mapping method includes four main steps described hereafter: (i) single-date multispectral classification into three classes, (ii) analysis of trajectory of changes using the temporal information and production of a “transition” map (with seven classes) (Figs. 1 and 2 and figs. S2 to S7), (iii) identification of subclasses of transition based on ancillary datasets (see Supplementary Text on ancillary datasets) and visual interpretation, and (iv) production of annual change maps (fig. S1).

In the first step, each image of the Landsat archive was analyzed on a single-date basis (through a multispectral classification), whereas previous large-scale studies used annual syntheses or intra-annual statistics, such as the mean and SD of available Landsat observations (*47*–*53*). The classification of individual images is challenging but presents three main advantages: it allows us (i) to capture the disturbance events that are visible from space for only a short period, such as logging activities; (ii) to record the precise timing of the disturbances and the number of disruption observations; and (iii) to detect the disturbance at an early stage, i.e., even if the disturbance starts at the end of the year, it is detected and counted as a disturbance for this year, whereas other approaches notably based on composites will detect the disturbance with a delay of 1 year.

A disruption observation is defined here as the detection of an absence of tree foliage cover within a Landsat pixel for a single-date observation. The number of disruption observations constitutes a proxy of disturbance intensity.

Each pixel within a Landsat image was initially assigned through single-date multispectral classification to one of the three following classes: (i) potential moist forest cover, (ii) potential disruption, and (iii) invalid observation (cloud, cloud shadow, haze, and sensor issue). The temporal sequence of classes (i) and (ii) was then used to determine the seven transition classes, described in the second step of the mapping approach. However, not all pixels could be unambiguously spectrally assigned to one of the three single-date classes because the multispectral cluster hulls of these classes are overlapping in the multidimensional feature space. In cases of spectral confusion, evidential reasoning was used to guide class assignment by taking into consideration the temporal trajectory of single-date classifications, as spectral overlap between land cover types may occur only during specific periods of the year. For instance, pixels covered by deciduous forests, grassland, or agriculture may behave, from a spectral point of view, as potential moist forest cover during the humid seasons and as potential disruptions during the dry seasons and, consequently, can be assigned to the other land cover transition class. Disturbed moist forests (degraded or deforested) appear as potential moist forest cover at the start of the archive and as potential disruption assignments later.

For the three initial classes (potential moist forest cover, potential disruption, and invalid observation), multispectral clusters were defined first by establishing a spectral library capturing the spectral signatures of the land cover types and atmosphere perturbations that are present over the pantropical belt and targeted for these three classes: (i) moist forest types; (ii) deciduous forest, logged areas, savanna, bare soil, irrigated and nonirrigated cropland, evergreen shrubland, and water (for the potential disruption class); and (iii) clouds, haze, and cloud shadows (for the invalid observations). A total sample of 38,326 sampled pixels belonging to 1512 Landsat scenes (L5, L7, and L8), were labeled through visual interpretation. The HSV (hue, saturation, and value) transformation of the spectral bands, well adapted for satellite image analysis (*44*, *54*), was used to complement the spectral library. These components were computed using a standard transformation (*55*) for the following Landsat band combination: short-wave infrared (SWIR2), near infrared, and red. The stability of hue to the impacts of atmospheric effect is particularly desirable for identifying potential disruption in the humid tropics. The sensitivity of saturation and value to atmospheric variability is mainly used to detect invalid observations (haze). Value is particularly useful for identifying cloud shadows. The thermal infrared band was relevant to detect invalid observations (clouds and haze) and bare soil and the normalized difference water index to identify irrigated areas. The information held in the spectral library was analyzed through visual analytics to extract equations describing class cluster hulls in the multidimensional feature space (fig. S17). An exploratory data analysis tool designed in a previous study (*44*) was used to support the interactive analysis.

In the second step of the mapping approach, the temporal sequence of single-date classifications at pixel scale was analyzed to first determine the initial extent of the TMF domain and then to identify the change trajectories from this initial forest extent (fig. S2). Long-term changes cannot be determined uniformly for the entire pantropical region because the observation record varies (see the “Data” section), e.g., the first year of observation (fig. S18) is c. 1982 for Brazil and c. 2000 along the Gulf of Guinea. We have addressed these geographic and temporal discontinuities of the Landsat archive by determining at the pixel level (i) a reference initial period (baseline) for mapping the initial TMF extent and (ii) a monitoring period for detecting the changes. The data gaps at the beginning of the archive were tackled by requiring a minimum period of 4 years with a minimum of three valid observations per year or a minimum of 5 years with two valid observations per year from the first available valid observation. Hence, the annual number of valid observations is lower, while the initial period is longer. This minimizes the risk of inclusion of nonforest cover types (such as agriculture) and deciduous forests in the baseline when there are few valid observations over a short period. In addition, we have reduced the commission errors in our baseline by accounting for possible confusion with commodities, wetlands, bamboo, and deciduous forest (see Supplementary Text on ancillary datasets and specific tropical forest types).

From our initial TMF extent, we identified seven main transition classes (fig. S2), which are defined thereafter. The first year of the monitoring period (which follows the initial period) is represented in fig. S18; it starts at the earliest in 1987 (mostly for South America) and, for very limited cases, at the latest in 2016 (e.g., Gabon).

Although no ecosystem may be considered truly undisturbed since some degree of human impact is present everywhere (*26*), we define the undisturbed moist forests (class 1) as tropical moist (evergreen or semievergreen) forest coverage without any disturbance (degradation or deforestation) observed over the Landsat historical record (see the “Study area and forest types” section). Our TMF baseline may include old forest regrowth (old secondary forests) or previously degraded forests, as the Landsat observation period is too short to distinguish never-cut primary forest from second-growth naturally recovered forest older than the observation period. This class includes two subclasses of bamboo-dominated forest (class 1a) and undisturbed mangrove (class 1b).

Deforested land (class 2) is defined as a permanent conversion from moist forest cover to another land cover, while a degraded forest (class 3) is defined as moist forest cover where disturbances were observed over a short period. Here, we assumed that the duration of the disturbance (and, consequently, the period over which we detect the disturbance with satellite imagery) is a proxy of the disturbance impact, i.e., the longer the duration of the detected disturbance, the higher is the impact on the forest and the higher the risk of having a permanent conversion of the TMF. In considering short-term disturbances, we include logging activities, fires, and natural damaging events, such as wind breaks and extreme dryness periods. Hence, we are getting closer to the most commonly accepted definition of degradation (*56*), one that considers a loss of productivity, a loss of biodiversity, unusual disturbances (droughts and blowdown), and a reduction of carbon storage.

The threshold applied to the duration parameter, used to separate degraded forests from deforested land, is based on our knowledge of impacts from human activity and from natural or human-induced events such as fires. We empirically identified two levels of degradation: (class 3a) degradation with short-duration impacts (observed within 1 year), which includes most logging activities, natural events, and minor fires; and (class 3b) degradation with long-duration impacts (from 1 to 2.5 years), which mainly corresponds to strong fires (burned forests). Most of the degradation (50%) is observed over a duration of less than 6 months (fig. S19). All disturbance events for which the impacts were observed over a period of more than 2.5 years (900 days) were considered as deforestation processes, with 68% of these events observed over more than 5 years. When a deforestation process is not followed by a regrowth period at least over the past 3 years, it is considered deforested land. Deforested land is also characterized by the recurrence of disruptions, i.e., the ratio between the number of years with at least one disruption observation and the total number of years between the first and last disruption observations. This information allowed us to discriminate between (i) deforestation of undisturbed forest and (ii) deforestation of degraded forest (when deforestation happens at least 1 year after degradation), with the second case (ii) having a lower recurrence of disruption events due to the period without any disruption between the degradation and deforestation stages (see Supplementary Text on annual change dataset).

For recent degradation and deforestation (class 4) that was initiated in the past 3 years (after year 2016) and that cannot yet be attributed to a long-term conversion to nonforest cover, owing to the limited historical period of observation, specific rules were applied. Within this class, we separated degradation from deforestation by taking a duration of at least 366 days for the years 2017–2018 and a threshold of 10 disruption observations for the past year (2019) to consider deforested land.

Forest regrowth (class 5) is a two-phase transition from moist forest to (i) deforested land and then (ii) vegetative regrowth. A minimum of 3-year duration of permanent moist forest cover presence is needed to classify a pixel as forest regrowth (to avoid confusion with agriculture).

Other land cover (class 6) includes savanna, deciduous forest, agriculture, evergreen shrubland, and nonvegetated cover. Last, vegetation regrowth (class 7) consists of a transition from other land cover to vegetation regrowth and includes two subclasses based on the age of regrowth (from 3 to 10 years and from 10 to 20 years) and a transition class from water to vegetation regrowth.

The third mapping step allowed us to identify three subclasses from the deforested land class. We geographically assigned deforestation to the following classes: conversion from TMF to tree plantations, mainly oil palm and rubber (class 2a); water surface (discriminating between permanent and seasonal water), mostly due to new dams (class 2b); and other land cover, such as agriculture, infrastructure, etc. (class 2c). This was carried out using ancillary spatial datasets completed by visual interpretation of high-resolution (HR) imagery (see Supplementary Text on ancillary data). Last, we reassigned disturbances within two geographically specific tropical forest formations: (i) the bamboo-dominated forest and (ii) the semideciduous transition tropical forest (see Supplementary Text on specific tropical forest formations).

Each disturbed pixel (degraded forest, deforested land, or forest regrowth) is characterized by the timing and intensity of the observed disruption events. The start and end dates of the disturbance allow us to identify, in particular, the timing of new road creation or of logging activities and the age of forest regrowth or degraded forests. Three decadal periods have been used in the transition map to identify age subclasses of degradation and forest regrowth: (i) before 2000, (ii) within 2000–2009, and (iii) within 2010–2019. The number of annual disruption observations combined with the duration can be used as a proxy for the disturbance intensity and impact level.

In the last mapping step, we created a collection of 30 maps providing the spatial extent of the TMF and disturbance classes on a yearly basis, from 1990 to 2019, using dedicated decision rules (see Supplementary Text on annual change dataset and thematic maps). These maps were used in our annual trend analysis, described in the next subsection, to document the annual disturbances over the full period, with 10 classes of transition for each annual statistic (Figs. 3 and 4 and figs. S1 and S11):

1) Short-duration degradation followed by a recovery period (not followed by deforestation).

2) Long-duration degradation followed by a recovery period (not followed by deforestation).

3) Degradation followed by a recovery period, followed by deforestation.

4) Degradation followed by a recovery period, followed by deforestation, and followed by a recovery period in itself (regrowth).

5) Deforestation of undisturbed forest, not followed by a recovery period.

6) Deforestation of undisturbed forest, followed by a recovery period (forest regrowth).

7) Deforestation of a degraded forest (with a recovery period between degradation and deforestation stages).

8) Deforestation of a degraded forest (with a recovery period between degradation and deforestation stages), followed by a recovery period (regrowth).

9) Deforestation with a forest conversion to water bodies.

10) Deforestation with a forest conversion to commodities.

To produce a more conservative map of undisturbed forests by excluding potential missed areas affected by logging activities, we created a disturbance buffer zone using a threshold distance of 120 m around disturbed pixels. This distance corresponds to the average observed distance between two logging decks (landing) and is consistent with the distances used in previous studies for assessing intact forests (*15*).

### Trend analysis

The areas of TMF and disturbance classes are reported annually in 5-year intervals from 1990 to 2019, by country, subregion, and continent (Tables 1 to 10, Figs. 3 and 4, fig. S11, and see Supplementary Text on trend analysis), using the country limits from the Global Administrative Unit Layers dataset from the FAO (*57*). Area measurements were also computed for 1° by 1° cells of a systematic latitude-longitude grid to delineate hot spot areas of deforestation and degradation for the three decades (Fig. 5). For the three most recent years of the considered period (i.e., for 2017–2019), the proportions of disturbance types (degradation followed by deforestation, degradation not followed by deforestation, and direct deforestation) were calibrated with historical proportions (2005–2014) of the three types of disturbances. For countries with moist forest areas larger than 5 million ha in 1990 (i.e., for 32 countries) and for all subregions, we analyzed the temporal dynamics of annual changes from 1990 to 2019 (fig. S11 and see Supplementary Text on trend analysis).

### Validation

The performance of our classifier was assessed in term of errors of omission and commission at the pixel scale, and the uncertainties in the area estimates derived from the transition map were quantified (see Supplementary Text on validation). A stratified systematic sampling scheme was used to create a reference dataset of 5250 sample plots of 3 by 3 pixels (0.81 ha of plot size) (fig. S8). For each sample plot, Landsat images on several dates were visually interpreted, together with the most recent HR images available from the Digital Globe or Bing collections, to create the reference dataset. The dates of the Landsat images to be interpreted were selected to optimize the assessment of the performance of our classifier as follows (fig. S9): (i) at least one random date within three successive key periods to verify the consistency of the temporal sequencing and the classifier performance across the main sensors (L5, L7, and L8); (ii) for the disturbed classes, the two dates corresponding to the first and last disruption observations were selected to assess the commission errors; and (iii) for the undisturbed forest class, at least one random date during the GFC loss year (if existing) to assess omission errors. It resulted in the interpretation of two to four Landsat images for each sample plot, with a total of 14,295 images.

The user, producer and overall accuracies, the confidence intervals of the estimated accuracies, and the corrected estimates of undisturbed and disturbed forest areas with a 95% confidence interval on this estimation were computed in accordance with the latest statistical practices (*27*). The performance of our disturbance detection results in 9.4% omissions, 8.1% false detections, and 91.4% overall accuracy (tables S2 and S3). In addition, the uncertainties of area estimates (forest cover and changes) have been assessed from a sample of 5119 reference plots. This accuracy assessment shows that a direct area measurement from the forest cover maps underestimates the forest area changes by 11.8% (representing 38.4 million ha, with 15 million ha having a confidence interval of 95%) (tables S4 and S5).

### Comparison with the GFC dataset

We compared our transition classes with the GFC dataset (*24*) for the TMF domain (undisturbed and degraded forest) in 2000 and over the period 2001–2019, which is the common period between the two products. We synthesized the GFC multiannual product into four classes of forest cover changes from the combination of the GFC annual layers of tree cover loss and gain over the period 2001–2019: (i) unchanged (no loss and no gain), (ii) at least one loss but no gain, (iii) at least one gain but no loss, and (iv) at least one loss and one gain. A new version of the transition map with eight classes was created (through the combination with annual maps) to characterize the disturbances that occurred from 2001 to 2019: (i) undisturbed forest (at the end of 2019), (ii) old degradation or regrowth (initiated before 2001), (iii) old deforestation (before 2001), (iv) degradation initiated from 2001 to 2019, (v) direct deforestation initiated from 2001 to 2019, (vi) deforestation that follows degradation and initiated from 2001 to 2019, (vii) regrowth initiated from 2001, and (viii) other land cover.

A matrix of correspondences between the synthesized GFC map (four classes) and our reclassified transition map (eight classes) was then produced for each continent and for the pantropical region, where area estimates are compared (table S1). This comparison shows that our annual change dataset depicts 138.9 million ha of forest disturbances in the period 2001–2019 that are not depicted in the GFC map (representing 59% of the total area of our disturbances). This finding is corroborated by previous studies (*32*, *33*). In addition, 17.6 million and 3.2 million ha are depicted as a GFC loss, whereas it is classified as old deforestation and degradation, respectively, (before 2001) in our TMF dataset. Among the disturbances that are not depicted by GFC, the greatest discrepancies concern the gradual processes such as degradation, the forest regrowth classes, and the deforestation that follows degradation. For these processes, the respective results showed that 75, 67, and 59% of our depicted areas are missing on the GFC map, whereas our direct deforestation class corresponds well with the GFC map (60%). The discrepancy between our dataset and the GFC map is even greater for the changes within mangroves, with an 83% difference. Mangroves are a key ecosystem within the TMF. We also observed a lower level of agreement for the disturbance classes in Africa (38% of our disturbances are depicted by GFC) compared with other continents (40.9 and 43.3% for Asia and Latin America, respectively). A higher underestimation of GFC loss in Africa compared with other continents has also been observed by Tyukavina *et al*. (*32*) using a sample-based analysis.

We observe greater discrepancies between GFC and our study for shorter and lower-intensity events. We observe an average duration of 6.7 years (visible impact) for the disturbances (both degradation and deforestation) detected only by our approach (and not by GFC) and an average duration of 9.4 years for the disturbances captured by both approaches (GFC and our approach). We observe an average intensity (or total number of disruptions detected for each disturbance) of 9.9 for the disturbances detected only by our approach compared with 32.6 for the disturbances captured by both approaches.

The evolution of the discrepancies over time shows major differences between the period (2001–2010) for which our annual change dataset depicts 61.4% more deforested areas and the past decade (2010–2019) for which GFC losses include all our deforestation areas and 5.7% of our degradation areas (Table 11 and Fig. 3). This change in the past decade has also been observed in another study (*58*) and can be explained (i) by the differences in processing applied by the GFC team before and after the year 2011 (https://earthenginepartners.appspot.com/science-2013-global-forest/download_v1.3.html) and (ii) by the inclusion of burned areas in the GFC loss (particularly for the dry period of 2015–2016) that are mainly classified as degradation in our TMF dataset.

### Projection of future forest cover

Temporal projections of future forest cover are provided for (i) undisturbed forest area and (ii) total forest area (undisturbed and degraded forests) per country (fig. S12 and table S8). We considered that the annual disturbed areas followed an independent log normal distribution for each country, and we used a modified version of the Cox method to estimate the means and the 95% confidence interval (*59*) of the distribution. We used these estimates for the past 10 years (period 2010–2019) to project disturbances over the period 2020–2050 under a business-as-usual scenario. Several metrics, with their uncertainties, have been produced: (i) forest area at the end of 2050, (ii) percentage of remaining forest area at the end of 2050 compared with forest area at the end of 2019, and (iii) year corresponding to full disappearance of forest cover.

### Known limitations and future improvements

Disturbances that affect less than the full pixel area (0.09-ha size), e.g., the removal of a single tree, are not included in our results when the impact of partial tree cover removal on the spectral values of the pixel is not strong enough to be detected. However, in specific cases, where the impact on the forest canopy cover significantly modifies the spectral values within a single pixel, e.g., the opening of a narrow logging road (<10 m wide) or the removal of several big trees, our approach can detect these disturbances.

We have addressed the geographic and temporal discontinuities of the Landsat archive (see the “Data” and “Mapping method” sections) by determining at the pixel level (i) an initial period (baseline) of at least 4 years (increasing when the annual number of valid observations is low) for mapping the initial TMF extent and (ii) a monitoring period for detecting the changes. This minimizes the risk of inclusion of nonforest cover types (such as agriculture) and deciduous forests in the baseline when there are few valid observations over a short period. This risk has been underestimated by previous studies that did not use a long period of analysis and did not account for the number of valid observations.

The accuracy of the disturbance detections has been assessed in the validation exercise (see the “Validation” section and Supplementary Text on validation). The assignment of the disturbance types at any location improves as the number of valid observations increases. The meta-information documents (i) the annual number of valid observations, (ii) the first year of valid observation (fig. S15), and (iii) the start year of the monitoring period (fig. S18) at each pixel location. This meta-information (in particular, the number of valid observations) can be considered as a proxy measure of confidence. Hence, our estimates of changes in the regions where the total number of valid observations is particularly low and/or the start year of the monitoring period is late (figs. S14, S15, and S18), e.g., Gabon, Solomon Islands, and La Réunion, should be considered with lower confidence. However, considering the geographic completeness of Landsat 8 coverage after the year 2013, there is high confidence for the contemporary reported estimates.

Short-duration events are likely to be underestimated for regions with geographic and temporal discontinuities in the Landsat archive and/or with gaps caused by persistent cloud cover. This is the case for Africa, which is poorly covered by Landsat acquisitions before the year 2000 (fig. S16). To provide a more conservative estimate of the remaining undisturbed forested areas, we also produced another estimate of undisturbed forested areas, using a buffer zone with a threshold distance of 120 m from the detected disturbed pixels to exclude the potentially edge-affected forest areas. Further contextual spatial analysis would be needed to better estimate the characteristics of fragmented areas.

At pantropical scale, with fine spatial resolution and annual frequency, detailed information about the historical forest area changes within the plantation concessions of oil palm and rubber are provided through to the combination of ancillary information and dedicated visual interpretation (see Supplementary Text on ancillary datasets). Although some confusion between forests and old plantations may remain (in particular, for plantations that are not included in the ancillary database of concessions or that cannot be easily identified visually in satellite imagery from a regular geometrical shape), these errors are expected to be limited because of the consideration of (i) a minimum duration for the initial period and (ii) a long observation period. Classes of tree plantations do not include all commodities, such as coffee, tea, and coconut, that are detected as deforested land (if initially TMF and converted into commodity during the monitoring period) or other land cover (if the concession was already established during the initial period).

Some isolated commission errors may remain in the bamboo-dominated TMF, wetlands, and semideciduous forests, as reference data were available on restricted areas (see Supplementary Text on specific tropical forest types). These will be continuously improved as the reference information layers improve and based on the feedback of users and national authorities.

The L7 SLC-off issue may introduce some spatial inconsistencies owing to a higher number of valid observations outside the SLC-off stripes, allowing more disruptions to be captured and, potentially, leading to a different transition class.

Efforts have been made to classify disturbances based on their characteristics (timing, recurrence, and sequence) to fit with the land cover use. However, all metrics used in this study are made freely available to the end user, who may apply different decision criteria that would better match specific user needs and constraints, e.g., the threshold applied to discriminate between deforestation and degradation may be different because of the selected definition of degradation.

This approach can be applied to future Landsat data (from 2020) automatically and is intended to be adapted to Sentinel-2 data (available since 2015) for TMF monitoring with higher temporal frequency and finer spatial resolution.

## SUPPLEMENTARY MATERIALS

Supplementary material for this article is available at http://advances.sciencemag.org/cgi/content/full/7/10/eabe1603/DC1
